# Missed opportunities for sexually transmitted infections testing for HIV pre‐exposure prophylaxis users: a systematic review

**DOI:** 10.1002/jia2.25673

**Published:** 2021-02-18

**Authors:** Jason J Ong, Hongyun Fu, Rachel C Baggaley, Teodora E Wi, Joseph D Tucker, M Kumi Smith, Sabrina Rafael, Jane Falconer, Fern Terris‐Prestholt, Ioannis Mameletzis, Phillipe Mayaud

**Affiliations:** ^1^ London School of Hygiene and Tropical Medicine London UK; ^2^ Monash University Melbourne Australia; ^3^ Eastern Virginia Medical School Norfolk VA USA; ^4^ World Health Organization Geneva Switzerland; ^5^ University of North Carolina at Chapel Hill Chapel Hill NC USA; ^6^ University of Minnesota Minneapolis MN USA

**Keywords:** sexually transmitted infections, HIV, pre‐exposure prophylaxis, sexual health, STI testing, systematic review

## Abstract

**Introduction:**

Given the synergistic relationship between HIV and sexually transmitted infections (STI), the integration of services has the potential to reduce the incidence of both HIV and STIs. We explored the extent to which STI testing has been offered within HIV pre‐exposure prophylaxis (PrEP) programmes worldwide.

**Methods:**

We conducted a systematic review of PrEP programmes implementing STI testing services in nine databases. We approached PrEP implementers for additional unpublished data and implementation details. Descriptive statistics were used to present the characteristics of STI testing within PrEP programmes. Content analysis of the input from PrEP implementers was conducted to summarize the barriers to and facilitators of STI testing.

**Results:**

Of 9,161 citations, 91 studies conducted in 32 countries were included: 69% from high‐income countries (HICs) and 64% from programmes targeting men who have sex with men (MSM) and transgender women (TGW) only. The majority of programmes (70%, 64/91) conducted STI testing before the initiation of PrEP. The most common STIs tested were gonorrhoea (86%, 78/91), chlamydia (84%, 76/91) and syphilis (84%, 76/91). The majority provided STI testing at three‐month intervals (70%, 53/76, for syphilis; 70% 53/78, for chlamydia; 68%, 53/78, for gonorrhoea). Relative to low‐ and middle‐income countries (LMICs), a higher proportion of PrEP programmes in HICs offered testing for gonorrhoea (92% vs. 71%, *p* < 0.05), chlamydia (92% vs. 64%, *p* < 0.01), syphilis (87% vs. 75%, *p* < 0.05), hepatitis A (18% vs. 4%, *p* < 0.05) and hepatitis C (43% vs. 21%, *p* < 0.05); offered testing for a higher number of STIs (mean 3.75 vs. 3.04, *p* < 0.05); and offered triple (throat, genital/urine and anorectal) anatomical site screening (54% vs. 18%, *p* < 0.001). Common implementation challenges included costs, access to STI diagnostics, programme logistics of integrating STI testing into PrEP delivery models and lack of capacity building for staff involved in PrEP provision.

**Conclusions:**

Significant gaps and challenges remain in the provision of STI testing services within HIV PrEP programmes. We recommend more active integration of STI testing and management into PrEP programmes, supported by standardized practice guidelines, staff capacity building training and adequate funding. This could lead to improved sexual health and HIV outcomes in key populations.

## 1 INTRODUCTION

Pre‐exposure prophylaxis (PrEP) is a safe and effective approach to prevent HIV infection when adherence is high [1, 2, 3, 4]. PrEP was first approved for use as an HIV prevention strategy in the United States in 2012, followed by 38 other countries/regions [5]. PrEP is offered in a total of 76 countries in various forms, including within research studies, clinical trials, demonstration projects or routine implementation, as of January 2021 [5]. With growing interest in PrEP, more members of key populations are engaging with healthcare systems than ever before. This provides a unique opportunity to package PrEP services with more comprehensive sexually transmitted infection (STI) testing, management and other sexual health services at a moment of peak receptivity, particularly in LMICs where such services are currently limited.

The World Health Organization (WHO) guidelines recommend that PrEP programmes target individuals at substantial risk for HIV [6], which overlap with populations at high risk of other STIs. There is a high prevalence of STIs among individuals initiating PrEP, and a high incidence of STIs in persistent users of PrEP [7]. The high incidence must be understood in light of the potential for ascertainment bias as more asymptomatic individuals will be screened for STIs as part of optimal PrEP care. These findings underscore the opportunity and need for active integration of HIV and STI testing and treatment services to tackle the synergistic epidemics of HIV/STI and capitalize on the existing and growing PrEP programme infrastructure globally.

To date, no study has addressed the extent to which STI testing services have been incorporated within PrEP services. We conducted a systematic review to describe the type of STI testing services offered to users of existing PrEP programmes worldwide. We supplemented these data with a survey of PrEP programme implementers regarding the facilitators and barriers to STI testing services for PrEP users. The findings from this study will provide important insights to inform the next steps of PrEP expansion and advocate for the need to integrate STI testing. Achieving this will contribute to the UN Sustainable Development Goals of ending the dual epidemics of HIV and STIs, and improving sexual health outcomes [8].

## 2 METHODS

This review was conducted in two stages and described in detail here [7]. Briefly, a systematic review was conducted in accordance with the PRISMA checklist (File S1) and registered (PROSPERO Registration: CRD42018116721). Second, supplemental data were collected between December 2018 and March 2019 through contacting a list of 82 PrEP programme implementers and/or researchers provided by the WHO and co‐authors to learn about their experiences in implementing PrEP service delivery on the ground.

The systematic review followed the guidelines in the Cochrane Handbook 5.1 [9]. Nine databases were searched from inception to 20 November 2018 (and updated on 8 December 2020) – as part of a WHO Think Tank Meeting for STIs and PrEP (March 2019) – without language restriction: OvidSP Medline and In‐Process & Other Non‐Indexed Citations and Daily, OvidSP Embase, OvidSP Global Health, OvidSP EconLit, EBSCO CINAHL Plus, EBSCO Africa‐Wide Information, Web of Science Core Collection, VHL LILACS and OvidSP Northern Light Life Sciences Conference Abstracts. The two key concepts anchoring our search strategy were STIs and PrEP, full details are published elsewhere, including our search terms [10]. File S2 provides details of the updated search. We included data from routine implementation programmes, prospective cohorts, randomized controlled trials (RCT) or demonstration projects of oral PrEP that described an STI testing service for PrEP users. We excluded systematic reviews/letters/editorials, studies using only qualitative research methods, duplicated results from the same study, laboratory studies about testing STI diagnostic performance and studies restricting study populations by clinical outcomes (e.g. men with urethritis, women with cervicitis). These studies were categorized as “wrong study design.” We manually searched the references of existing systematic reviews [11, 12, 13] to ensure our search strategy included all relevant papers. Once duplicates were removed, the titles and abstracts of papers were independently screened by at least two reviewers (HF, SR), according to a list of eligibility criteria, and disagreements were discussed with author JO. Two researchers (HF, SR) extracted data from the full texts, and data were reviewed by JO for consistency and accuracy. Descriptive statistics (mean continuous variables and percentages for categorical variables) were used to present the characteristics of STI services within PrEP programmes.

To obtain additional unpublished data and more detailed information on PrEP implementation, we contacted 82 PrEP implementers (list provided by WHO of contacts of known PrEP programmes) by e‐mail with an invitation to complete a survey questionnaire that included both closed‐ended and open‐ended questions between December 2018 and April 2019 (File S3). A follow‐up email reminder was sent 7 days later if there had not been any response. We present descriptive statistics to summarize the characteristics of the PrEP programmes (this term will be used to include both data from studies and programmes) included in this review, including the characteristics of PrEP programmes and sites, by country income‐level, project implementation duration, target population, PrEP service sites, study/programme design as well as the provision of STI testing and additional sexual health services. Differences in STI testing by PrEP programme characteristics were analysed using chi‐square test for the categorical outcomes (e.g. whether or not specific STI testing was provided) and T‐test for the continuous outcome variables (e.g. number of STIs tested). A *p* < 0.05 was defined as statistically significant. Statistical analyses were performed using STATA version 16 (StataCorp. 2019. *Stata Statistical Software: Release 16*. College Station, TX: StataCorp LLC). A content analysis of reports from the PrEP implementers was used to summarize the challenges and facilitators to provide STI testing to PrEP users. As this review was focused on describing STI services for PrEP users, no risk of bias assessment was conducted.

No ethics approval was needed as this was a systematic review with no patient involvement. Consent was implied if PrEP providers responded to providing information about their PrEP programme.

## 3 RESULTS

**Figure 1:**
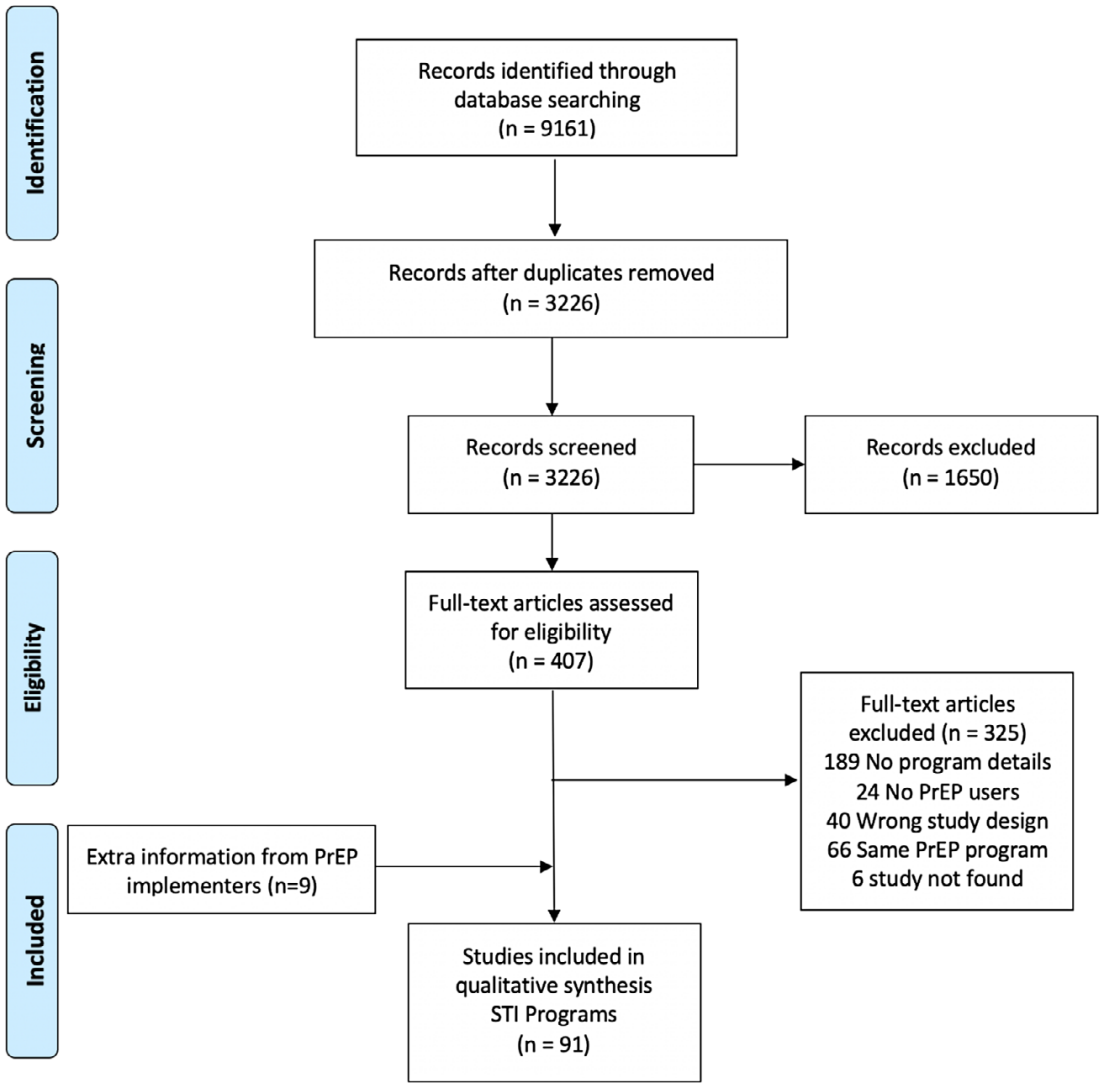
PRISMA flow chart.

Of 9161 citations identified, 82 publications and nine unpublished data from PrEP implementers were included in this analysis (Figure 1). Table 1 summarizes the characteristics of these 91 PrEP programmes. Data came from 32 countries, mostly from high‐income countries (HICs) (63/91, 69%), including 40 programmes (44%) from the United States alone. The majority of the programmes were targeting men who have sex with men (MSM) and transgender women (TGW) only (64%, 58/100). Around half of the PrEP programmes were delivered through hospital‐based outpatient facilities including sexual health clinics (50/91, 55%). More details from these programmes are reported in Table S1.

**Table 1:** Characteristics of STI testing services within PrEP programmes (N = 91)

Programme indicators	n (%)
Country income level	
High‐income	63 (69%)
Low‐ or middle‐income	28 (31%)
First year of data	
Before 2013	13 (14%)
2013 to 2015	31 (34%)
After 2016	47 (52%)
PrEP users population	
MSM/TGW	58 (64%)
Mixed population^a^	33 (36%)
Programme services site	
Hospital and sexual health clinic	50 (55 %)
A mix of hospital and community clinic	20 (22%)
Community‐based organizations/settings	18 (20%)
General practice	3 (3%)
Type of study	
Routine implementation	28 (31%)
Open‐label cohort study	39 (43%)
Demonstration project	17 (19%)
Randomized controlled trial	7 (8%)
Provided STI testing before PrEP initiation	
Yes	64 (70%)
Not stated	27 (30%)
Provided gonorrhoea testing	
Yes	78 (86%)
Not stated	13 (14%)
Gonorrhoea testing frequency (N = 78)	
Every three months or shorter interval	53 (68%)
Every six months	17 (22%)
Every twelve months or longer	4 (5%)
Not stated	4 (5%)
Gonorrhoea testing methods (N = 78)	
Molecular testing	56 (72%)
Not stated	22 (28%)
Provided chlamydia testing	
Yes	76 (84%)
Not stated	15 (16%)
Chlamydia testing frequency (N = 76)	
Every three months or shorte	53 (70%)
Every six months	17 (22%)
Every twelve months or longer	4 (5%)
Not stated	2 (3%)
Provided syphilis testing	
Yes	76 (84%)
Not stated	15 (16 %)
Syphilis testing frequency (N = 76)	
Every three months or shorter	53 (70%)
Every six months	15 (20%)
Every twelve months or longer	5 (6%)
Not stated	3 (4%)
Syphilis testing method (N = 76)	
Serology	41 (54%)
Rapid diagnostic test	11 (14%)
PCR	2 (3%)
Not stated	22 (29%)
Provided Hepatitis A testing	
Yes	12 (13%)
Not stated	79 (87%)
Hepatitis A testing frequency (N = 12)	
At baseline only	6 (50%)
Every three months	5 (42%)
Every twelve months	1 (8%)
Provided Hepatitis A vaccination	
Yes	8 (9%)
Not stated	83 (91%)
Provided Hepatitis B testing	
Yes	48 (53%)
Not stated	43 (47%)
Hepatitis B testing frequency (N = 48)	
Every three months	14 (29%)
Every six months	2 (4%)
Every twelve months	5 (10%)
At baseline only	18 (38%)
Not stated	9 (19%)
Provided Hepatitis B vaccination	
Yes	17 (19%)
Not stated	74 (81%)
Provided Hepatitis C testing	
Yes	33 (36%)
Not stated	58 (64%)
Hepatitis C testing frequency (N = 33)	
Every three months	10 (30%)
Every six months	5 (15 %)
Every twelve months	6 (18%)
At baseline only	5 (15%)
Not stated	7 (22%)
Provided human papillomavirus vaccination	
Yes	5 (6%)
Not stated	86 (94 %)
Number of STIs tested^b^ (Range: 0 to 6)	
Mean (SD)	3.53 (1.59)
Median (interquartile range)	3 (3 to 5)
Provided additional sexual health services^c^	
Yes	48 (53%)
Not stated	43 (47%)
Triple anatomical site screening available^d^	
Yes	39 (43%)
Not stated	52 (57%)
Provided antimicrobial resistance testing	
Yes	7 (8%)
Not stated	84 (92%)

MSM, men who have sex with men; SD, standard deviation; STI, sexually transmitted infection; TGW, transgender women.

The above notes also apply to Tables 2, 3, 4.

a Mixed population included sero‐discordant couples, female sex workers, cis‐gender females, transgender men, people who use drugs and heterosexuals

b types of STIs tested included gonorrhoea, chlamydia, syphilis, Hepatitis A, Hepatitis B, Hepatitis C

c additional sexual health services include vaccination, free condoms, counselling, partner notification services, etc.

d triple site screening availability is defined as whether the use of throat, urethra/urine and anorectal samples were available at the clinic.

The majority of programmes (70%, 64/91) conducted STI testing before the initiation of PrEP. The most common STIs tested were gonorrhoea (86%, 78/91), chlamydia (84% 76/91) and syphilis (84% 76/91). The majority of programmes provided routine testing for those three STIs at three‐month intervals (70%, 53/76, for syphilis; 70% 53/78, for chlamydia; 68%, 53/78, for gonorrhoea). Molecular testing (72%, 56/78) was the most commonly reported method used for chlamydia and gonorrhoea testing; and serology for syphilis testing. A proportion of the programmes also reported the testing of Hepatitis B (53%, 48/91), Hepatitis C (36%, 33/91) and Hepatitis A (13%, 12/91) with testing mainly provided only at baseline (50%, 6/12, for Hepatitis A, 38%18/48, for Hepatitis B, 15%, 5/33 for Hepatitis C), using serological laboratory‐based tests. On average, more than three STIs were tested in the PrEP programmes (mean 3.53; SD 1.59). A small proportion of programmes also provided vaccination for Hepatitis B (19%, 17/91), Hepatitis A (9%, 8/91), or human papillomavirus (HPV) (5%, 5/91). More than half of PrEP programmes (53%, 48/91) provided additional sexual health services (e.g. condom/lubricants distribution, HIV/STI risk assessment, safe sex counselling, risk reduction and health education counselling etc.). Triple anatomical sites (throat, urethra/urine, anorectal) screening was available in 43% (39/91) of PrEP programmes and 48% (28/58) of PrEP programmes targeting MSM/TGW. Antimicrobial resistance testing was reported in 8% (7/91) of the programmes.

Table 2 reports the differences in STI testing by the income level of the country where PrEP programmes were implemented. Relative to low‐and middle‐income countries (LMICs), programmes in HICs significantly had a higher availability of testing for gonorrhoea (92% vs. 71%, *p* < 0.05), chlamydia (92% vs. 64%, *p* < 0.01), syphilis (87% vs. 75%, *p* < 0.05), hepatitis A (18% vs. 4%, *p* < 0.05), hepatitis C (43% vs. 21%, *p* < 0.05), more frequent testing (three‐month interval) for gonorrhoea (72% vs. 45%, *p* < 0.08, marginally significant) and chlamydia (72% vs. 45%, *p* < 0.09, marginally significant), being tested for a higher number of STIs (mean 3.75 vs. 3.04, *p* < 0.05), and offered triple anatomical site STI testing (54% vs. 18%, *p* < 0.001).

**Table 2:** Differences in STI testing services in PrEP programmes by the income levels of countries where PrEP programmes were implemented (N = 91)

Programme indicators	Country income level		*p*‐value
	LMIC	HIC	
	n (%) (N = 28)	n (%) (N = 63)	
Had STI testing before PrEP initiation			
Yes	21 (74%)	43 (68%)	0.35
Not stated	7 (26%)	20 (32%)	
Provided gonorrhoea testing			
Yes	**20 (71%)**	**58 (92%)**	**0.01**
Not stated	**8 (29%)**	**5 (8%)**	
Gonorrhoea testing frequency (N = 78)			
Every three months	9 (45%)	42 (72%)	0.08
Longer than three months interval	9 (45%)	14 (24%)	
Not stated	2 (10%)	2 (3%)	
Provided chlamydia testing			
Yes	**18 (64%)**	**58 (92%)**	**0.002**
Not stated	**10 (36%)**	**5 (8%)**	
Chlamydia testing frequency (N = 76)			
Every three months	9 (45%)	42 (72%)	0.09
Longer than every three months	9 (55%)	14 (24%)	
Not stated	0 (0%)	2 (4%)	
Provided syphilis testing			
Yes	**21 (75%)**	**55 (87%)**	**0.04**
No stated	**7 (25%)**	**8 (13%)**	
Syphilis testing frequency (N = 76)			
Every three months	13 (62%)	40 (73%)	0.23
Longer than every three months	8 (38%)	12 (22%)	
Not stated	0 (0%)	3 (6%)	
Provided hepatitis A testing			
Yes	**1 (4%)**	**11 (18%)**	**0.05**
Not stated	**27 (96%)**	**52 (82%)**	
Hepatitis A testing frequency (N = 12)			
Every three months	1 (100%)	6 (55%)	0.5
At baseline only	0 (0%)	5 (45%)	
Provided hepatitis A vaccination			
Ye	1 (4%)	7 (11%)	0.23
Not stated	27 (96%)	56 (89%)	
Provided hepatitis B testing			
Yes	15 (54%)	33 (52%)	0.55
Not stated	13 (46%)	30 (48%)	
Hepatitis B testing frequency (N = 48)			
Testing at every 3/6/12 months	7 (47%)	14 (42%)	0.3
At baseline only	7 (47%)	11 (33%)	
Not stated	1 (6%)	8 (24%)	
Provided Hepatitis B vaccination			
Yes	5 (31%)	12 (19%)	0.57
Not stated	23 (69%)	51 (81%)	
Provided Hepatitis C testing			
Yes	**6 (21%)**	**27 (43%)**	**0.04**
Not stated	**22 (79%)**	**36 (57%)**	
Hepatitis C testing frequency (N = 33)			
Testing at every 3/6/12 months	4 (67%)	17 (63%)	0.32
At baseline only	2 (33%)	3 (11%)	
Not stated	0 (0%)	7 (26%)	
Provided HPV vaccination			
Ye	1 (4%)	4 (6%)	0.48
Not stated	27 (96%)	59 (94%)	
Numbers of STIs tested^b^ (Range: 0 to 6)			
Mean (SD)	**3.04 (1.60)**	**3.75 (1.56)**	**0.04**
Additional sexual health services provided			
Yes	16 (57%)	32 (51%)	0.37
Not stated	12 (43%)	31 (49%)	
Triple anatomical site STI screening available^d^			
Yes	**5 (18%)**	**34 (54%)**	**0.001**
Not stated	**23 (82%)**	**29 (46%)**	
Provided antimicrobial resistance testing			
Yes	2 (7%)	5 (8%)	0.63
Not stated	26 (93%)	58 (92%)	

Please refer to the notes under Table 1 for the definitions of variables and abbreviations Regarding statistical analyses, student T test was used to examine the difference in number of STI tested (continuous variable) between HICs and LMIC. We used the Chi‐square test for categorical variables. The same types of analyses were used to produce the results in Tables 3 and 4. Numbers in bold indicate a statistically significant difference (*p* < 0.05).

Table 3 reports the differences in STI testing by the characteristics of PrEP programme design. Results revealed a significant difference in the frequency of STI testing before PrEP initiation, with the rate being the highest for RCT study (86%), followed by cohort study (82%), demonstration project (77%) and routine implementation (46%) (*p* < 0.05). On average, routine implementation project (81%) had the highest frequency (three monthly) for syphilis testing, followed by cohort study (69%), demonstration project (67%) and RCT study (29%) (*p* < 0.05). The frequency of Hepatitis B testing was the highest in demonstration project (83%), followed by routine implementation (50%), cohort study (45%) and RCT study (29%) (*p* < 0.05).

**Table 3:** Differences in STI testing services by PrEP programme study design (N = 91)

Programme indicators	Type of PrEP programme				*p*‐value
	Routine implementation	Cohort Study	Demonstration project	RCT Study	
	n (%) (N = 28)	n (%) (N = 39)	n (%) (N = 17)	n (%) (N = 7)	
Had STI testing before PrEP initiation					
Yes	**13 (46%)**	**32 (82%)**	**13 (77%)**	**6 (86%)**	**0.01**
Not stated	**15 (53%)**	**7 (18%)**	**4 (23%)**	**1 (14%)**	
Provided gonorrhoea testing					
Yes	24 (86%)	34 (87%)	14 (82%)	6 (86%)	0.98
Not stated	4 (14%)	5 (13%)	3 (18%)	1 (5%)	
Gonorrhoea testing frequency (N = 78)					
Every three months	17 (71%)	24 (71%)	8 (57%)	2 (33%)	0.23
Longer than three months interval	5 (21%)	9 (29%)	5 (36%)	4 (67%)	
Not stated	2 (8%)	1 (3%)	1 (7%)	0 (0%)	
Provided chlamydia testing					
Yes	24 (86%)	33 (85%)	13 (77%)	6 (86%)	0.86
Not stated	4 (14%)	6 (15%)	4 (23%)	1 (14%)	
Chlamydia testing frequency (N = 76)					
Every three months	17 (71%)	24 (73%)	8 (57%)	2 (33%)	0.16
Longer than every three months	5 (21%)	9 (27%)	5 (43%)	4 (67%)	
Not stated	2 (8%)	0 (0%)	0 (0%)	0 (0%)	
Provided syphilis testing					
Yes	24 (86%)	32 (82%)	13 (77%)	7 (100%)	0.54
No stated	4 (14%)	7 (18%)	4 (23%)	0 (0%)	
Syphilis testing frequency (N = 76)					
Every three months	**16 (67%)**	**26 (81%)**	**9 (69%)**	**2 (29%)**	**0.02**
Longer than every three months	**5 (21%)**	**6 (19%)**	**4 (31%)**	**5 (71%)**	
Not stated	**3 (12%)**	**0 (0%)**	**0 (0%)**	**0 (0%)**	
Provided Hepatitis A testing					
Yes	4 (14%)	5 (13%)	2 (11%)	1 (14%)	0.99
Not stated	24 (86%)	34 (87%)	15 (89%)	6 (86%)	
Hepatitis A testing frequency (N = 12)					
Every three months	1 (25%)	4 (80%)	1 (50%)	0 (0%)	0.28
At baseline only	3 (75%)	1 (20%)	1 (50%)	1 (100%)	
Provided hepatitis A vaccination					
Yes	4 (14%)	1 (3%)	2 (12%)	1 (14%)	0.33
Not stated	24 (86%)	38 (97%)	15 (88%)	6 (86%)	
Provided Hepatitis B testing					
Yes	**14 (50%)**	**18 (45%)**	**15 (83%)**	**2 (29%)**	**0.01**
Not stated	**14 (50%)**	**22 (55%)**	**3 (17%)**	**5 (71%)**	
Hepatitis B testing frequency (N = 48)					
Testing at every 3/6/12 months	3 (21%)	7 (41%)	10 (77%)	1 (50%)	0.31
At baseline only	8 (57%)	6 (35%)	3 (23%)	1 (50%)	
Not stated	3 (21%)	4 (24%)	2 (14%)	0 (0%)	
Provided hepatitis B vaccination					
Yes	5 (18%)	5 (13%)	6 (35%)	1 (14%)	0.25
Not stated	23 (82%)	34 (87%)	11 (65%)	6 (86%)	
Provided hepatitis C testing					
Yes	11 (39%)	13 (33%)	8 (47%)	1 (14%)	0.46
Not stated	17 (61%)	26 (67%)	9 (53%)	6 (86%)	
Hepatitis C testing frequency (N = 33)					
Testing at every 3/6/12 months	8 (73%)	9 (69%)	4 (50%)	0 (0%)	0.28
At baseline only	1 (9%)	1 (8%)	2 (25%)	1 (100%)	
Not stated	2 (18%)	3 (23%)	2 (25%)	0 (0%)	
Provided HPV vaccination					
Yes	3 (11%)	0 (0%)	2 (12%)	0 (0%)	0.14
Not stated	25 (89%)	39 (100%)	15 (88%)	7 (100%)	
Numbers of STIs tested^b^ (Range: 0 to 6)					
Mean (SD)	3.36 (1.85)	3.67(1.40)	3.59 (1.73)	3.29 (1.38)	0.85
Additional sexual health services provided					
Yes	15 (54%)	19 (49%)	11 (65%)	3 (43%)	0.68
Not stated	13 (46%)	20 (51%)	6 (35%)	4 (57%)	
Triple anatomical site STI screening available^d^					
Yes	14 (50%)	15 (39%)	5 (29%)	5 (70%)	0.21
Not stated	14 (50%)	24 (61%)	12 (71%)	2 (30%)	
Provided antimicrobial resistance testing					
Yes	1 (4%)	4 (10%)	1 (6%)	1 (14%)	0.67
Not stated	27 (96%)	35 (90%)	16 (94%)	6 (86%)	

Please refer to the notes under Tables 1 and 2 regarding the definitions of variables and abbreviations, and statistical analyses used to produce the results. Numbers in bold indicate a statistically significant difference (*p* < 0.05).

Table 4 reports the differences in STI testing by PrEP programme participants. Compared to programmes which served mixed populations, a higher proportion of programmes which focused on MSM/TGW offered gonorrhoea, chlamydia syphilis testing and more sexual health services, although those differences did not reach statistically significant level.

**Table 4:** Differences in STI testing services in PrEP programmes by participant types (N = 91)

Programme indicators	Programme target populations		*p*‐value
	MSM/TGW	Mixed population^a^	
	n (%) (N = 58)	n (%) (N = 33)	
Had STI testing before PrEP initiation			
Yes	41 (71%)	23 (70%)	0.55
Not stated	17 (29%)	10 (30%)	
Provided gonorrhoea testing			
Yes	52 (90%)	26 (79%)	0.13
Not stated	6 (10%)	7 (21%)	
Gonorrhoea testing frequency (N = 78)			
Every three months	36 (69%)	15 (58%)	0.46
Longer than three months interval	13 (25%)	10 (39%)	
Not stated	3 (6%)	1 (4%)	
Provided chlamydia testing			
Yes	51 (88%)	25 (76%)	0.11
Not stated	7 (12%)	8 (24%)	
Chlamydia testing frequency (N = 76)			
Every three months	36 (71%)	15 (60%)	0.30
Longer than every three months	13 (25%)	10 (40%)	
Not stated	2 (4%)	0 (0%)	
Provided syphilis testing			
Yes	49 (85%)	27 (82%)	0.48
No stated	9 (15%)	6 (18%)	
Syphilis testing frequency (N = 76)			
Every three months	32 (65%)	21 (78%)	0.32
Longer than every three months	14 (29%)	6 (22%)	
Not stated	3 (6%)	0 (0%)	
Provided Hepatitis A testing			
Yes	8 (14%)	4 (12%)	0.55
Not stated	50 (86%)	29 (88%)	
Hepatitis A testing frequency (N = 12)			
Every three months	4 (50%)	2 (50%)	0.73
At baseline only	4 (50%)	2 (50%)	
Provided hepatitis A vaccination			
Yes	7 (12%)	1 (3%)	0.14
Not stated	51 (88%)	32 (97%)	
Provided hepatitis B testing			
Yes	29 (50%)	19 (58%)	0.32
Not stated	29 (50%)	14 (42%)	
Hepatitis B testing frequency (N = 48)			
Testing at every 3/6/12 months	13 (45%)	8 (42%)	0.32
At baseline only	10 (35%)	8 (42%)	
Not stated	6 (21%)	3 (16%)	
Provided hepatitis B vaccination			
Yes	13 (22%)	4 (12%)	0.18
Not stated	45 (78%)	29 (88%)	
Provided hepatitis C testing			
Yes	22 (37%)	11 (32%)	0.42
Not stated	37 (63%)	22 (68%)	
Hepatitis C testing frequency (N = 33)			
Testing at every 3/6/12 months	12 (71%)	9 (100%)	0.18
At baseline only	5 (29%)	0 (0%)	
Not stated	5 (23%)	2 (18%)	
Provided HPV vaccination			
Yes	5 (9%)	0 (0%)	0.10
Not stated	53 (91%)	33 (100%)	
Numbers of STIs tested^b^ (Range: 0 to 6)			
Mean (SD)	3.61 (1.63)	3.48 (1.58)	0.73
Additional sexual health services provided			
Yes	29 (50%)	19 (58%)	0.32
Not stated	29 (50%)	14 (41%)	
Triple anatomical site STI screening available^d^			
Yes	28 (48%)	11 (33%)	0.12
Not stated	30 (51%)	22 (67%)	
Provided antimicrobial resistance testing			
Yes	5 (9%)	2 (6%)	0.50
Not stated	53 (91%)	31 (94%)	

Please refer to the notes under Tables 1 and 2 regarding the definitions of variables and abbreviations, and statistical analyses used to produce the results in the tables.

A total of 21 implementers sent back their completed survey (response rate 27%) of whom 12 (seven from LMICs) also provided comments about the implementation challenges of providing STI services. There was a wide spectrum of STI services. On one end was the provision of PrEP services that were integrated into existing sexual health services (e.g. UK, Australia, USA, France). These programmes usually provided triple‐anatomical site screening and provided other services such as vaccinations for hepatitis A, hepatitis B and HPV, partner notification services, counselling, mental health and substance use support. There were often no direct user fees for PrEP users to utilize STI services as these were paid for by national governments (Australia, UK), insurance companies (USA) or philanthropic organizations (USA). In the middle of the spectrum were programmes which primarily dispensed PrEP but also had additional range of STI services (e.g. Vietnam, Japan, Brazil, Thailand, South Africa, Kenya). These were usually fee‐based services for STI consultations, testing and treatment, though in some cases a proportion of patient costs were subsidized by research funds or other external funds. Therefore, in these countries, only limited STI screening was available: syphilis testing only (South Africa) or chlamydia/gonorrhoea molecular testing only available for limited anatomical sites (urethral only in Vietnam, rectal swabs only in Malaysia, rectal and pharyngeal swabs in Japan). At the other end of the spectrum were PrEP programmes which did not have access to STI diagnostics and relied on syndromic management (e.g. Kenya, South Africa, Morocco).

### 3.1 Implementation challenges for provision of STI services to PrEP users

The main themes related to STI diagnostics, programme logistics of PrEP delivery and lack of STI capacity building. Regarding diagnostics, PrEP implementers from both HIC and LMIC settings discussed the lack of accurate, affordable and easy to use point‐of‐care STI tests for chlamydia/gonorrhoea. Although a near‐patient test (e.g. GeneXpert platform) is available, it remains unaffordable for many LMIC settings. Regarding PrEP service delivery, individuals described community‐based delivery models to improve PrEP access (e.g. Thailand, Kenya), but the decentralization of services brought unforeseen challenges around STI services (e.g. need for injectable antibiotics for syphilis/gonorrhoea treatment). Most managers from LMIC settings cited poor integration of STI services into PrEP programmes and perceived time constraints for PrEP providers to also discuss STI testing and management with PrEP users. Related to this was the lack of capacity building for PrEP providers to deliver STI services including the lack of basic equipment for vaginal or anal examinations and limited STI training for PrEP providers.

The main themes related to improving the provision of STI services included greater funding of programmes, better training and access to STI point‐of‐care tests. Regarding finances, the need for lower costs of STI diagnostics was brought up by all implementers. One implementor suggested the need for an international or regional bulk‐purchasing mechanism for STI diagnostics for sustainable supply, as exists for HIV diagnostics. Some stressed that funding should also be made available for provision of other sexual health services as well as for vaccinations, mental health and substance use support. Regarding training, one implementer suggested incorporating mandatory STI training for PrEP providers. It was noted that each country has their own testing protocols, with a need for clearer and more unified recommendation for STI testing in PrEP users by the WHO, particularly regarding the frequency of testing, which STIs to test, and what anatomical sites to test [14]. Regarding diagnostics, some made suggestions to consider pooled testing (i.e. grouping samples from various anatomical sites of the same patient into one sample), and STI self‐sampling or self‐testing. More research was suggested to develop and evaluate accurate, affordable easy to use POCT for STI diagnosis and simpler, effective treatment regimens (oral preferred).

## 4 DISCUSSION

Given the high prevalence and incidence of STIs among PrEP users reported in a previous systematic analysis across several settings [7], regular STI testing is recommended for PrEP users. However, this systematic review highlights the missed opportunities in providing STI testing within PrEP programmes. PrEP has been effective in reducing HIV at the population level [15, 16], and if STI services can be integrated within all PrEP programmes, there is the potential to also control STI pandemics [17]. Higher coverage and more frequent STI testing among high‐risk individuals using PrEP and their partners could reduce STI prevalence [18, 19].

Globally, STI services are often underprioritized and underfunded, particularly in resource‐limited settings. We identified a spectrum of STI services implemented in PrEP programmes with key differences according to country income level, type of PrEP programme and target population. Given the high STI prevalence at entry into the programme and STI incidence during PrEP use [20], additional STI services could improve value for money for these services. With increasing demand for PrEP globally, this is an opportunity to ensure more comprehensive STI and HIV service integration to interrupt the chains of STI transmission among sexual networks with high prevalence and incidence of STIs [7]. This synergistic approach improves existing health inequities and inefficiencies from a public health perspective [21], particularly if PrEP programmes can be the gateway for more comprehensive sexual health services for PrEP users: including better access to condoms and lubricants, reproductive choices, vaccinations for hepatitis A and B and HPV and substance use and mental health support.

Our review revealed challenges in implementing integrated STI services within PrEP programmes. The key challenges raised by PrEP implementers included: (1) limited resources for STI testing; (2) programme logistics of combined STI and PrEP delivery and (3) inadequate capacity and training of programme staff to deliver STI services to PrEP users. The lack of resources for STI molecular diagnostics (particularly limited options of POC testing for chlamydia and gonorrhoea) [22] has hampered many countries’ capacity to provide aetiological STI diagnoses, with greater reliance on syndromic management or limited use of diagnostics. This results in substantial missed and over treatment, poor antibiotic stewardship and the inability to control STIs, as the majority of individuals with an STI are asymptomatic and antibiotic resistant‐STIs could be concentrated within specific sexual networks [23]. As PrEP programmes continue to be scaled up globally, we recommend greater attention and resource allocation towards addressing the logistics of offering STI services and STI training of staff delivering PrEP. In particular, most PrEP users currently are MSM/TGW in HIC but there is increased interest and funding available for PrEP in LMIC for MSM/TGW and for other groups at substantial HIV risk such as sex workers young women and couples in Eastern and Southern Africa, in where there is an urgent need for incorporating sexual and reproductive health services (e.g. family planning, contraceptives and pregnancy testing) [24]. We also need greater access to practical implementation lessons from other PrEP programmes that have successfully integrated HIV/STI testing [25].

Increasing access to STI services among PrEP users has the potential for beneficial direct and indirect spillover effects for non‐PrEP users. Direct benefits include earlier diagnosis and management of STIs among PrEP users, which can reduce the community burden of STIs [18]. Indirect benefits include the strengthening of a country’s infrastructure of sexual health services which can benefit the community’s sexual and reproductive health as a whole. To ensure equitable access to PrEP for all subpopulations, there must be continued efforts to diversify how PrEP can be accessed – not just through medical facilities [25].

The strengths of our review are the inclusion of data from 32 countries including non‐MSM populations, inclusion of several LMIC settings, with first‐hand supplemental data collected directly from PrEP implementers worldwide. Our findings should be considered in light of several limitations: (1) There were inconsistencies in documentation of STI testing in existing PrEP programmes. The missing information on STI testing in PrEP programmes could potentially result in either an underestimation of STI testing for PrEP users or over‐estimation if only PrEP programmes with STI services responded to the survey. Furthermore, there was sparse information on linkage to appropriate management once an individual was diagnosed with an STI. This was beyond the scope of our study but future research to confirm linkage to care is happening. (2) We grouped findings from research studies and routinely implemented programmes; we acknowledge that there are different funding constraints depending on the type of PrEP programmes, which may affect the feasibility of providing STI testing. (3) We had limited power to conduct multivariable analysis to ascertain the differences between STI testing across PrEP programmes. Many results on the differences in STI testing across PrEP programmes were statistically insignificant at bi‐variable analysis, although many of the differences were significant in size. Future studies (including stakeholder analyses) exploring the differences between STI testing programmes for PrEP users would be beneficial. (4) We did not find any economic evaluations of incremental costs related to integrating STI services within PrEP programmes. This is an area for future research to evaluate the potential for economies of scope and scale, and would support advocacy for the active integration of sexual health services within PrEP programmes. (5) Many studies came from the United States which may skew our findings presented in Table 2.

## 5 CONCLUSIONS

This review documents the integration of STI services within existing PrEP programmes globally. Overall, there is inconsistent access to STI services for PrEP users with programmes testing a different number of STI pathogens with different frequencies and variable integration of other sexual health and preventative services (e.g. vaccination). Together, the evidence suggests the need for more comprehensive STI services available to PrEP users. In particular, with the rising interest among LMIC in expanding PrEP among key populations with overlapping risk factors for STIs, this represents a unique opportunity to facilitate integrated HIV and STI service models, which will contribute to curtailing the synergistic epidemics of HIV and STIs in high‐risk underserved populations globally.

## COMPETING INTEREST

All authors declare that they do not have any conflict of interest.

## AUTHORS’ CONTRIBUTIONS

JJO, PM, RB and TW designed the research study. JJO, HYF, MKS, SR and JF performed the research. JJO and HYF analysed the data. All authors (JJO, HYF, RCB, TEW, JDT, MKS, SF, JF, FTP, IM and PM) contributed to writing the paper and approved the final manuscript for submission.

## Supporting information


**Table S1.** PREP studies included in the systematic review (N = 91).
**File S1.** PRISMA checklist
**File S2.** Search methodology and search results
**File S3.** Survey for PrEP implementers
