# Supplementary material for: Missed opportunities for sexually transmitted infections testing for HIV pre‐exposure prophylaxis users: a systematic review
Source: J Int AIDS Soc. 2021 Feb 18;24(2):e25673. doi: 10.1002/jia2.25673 (PMC7893146; doi:10.1002/jia2.25673)
Supplement: Supplementary file 1 — Table S1. PREP studies included in the systematic review (N = 91). File S1. PRISMA checklist File S2. Search methodology and search results File S3. Survey for PrEP implementers [file JIA2-24-e25673-s001.docx]

**APPENDICES**

**Supplemental Table 1: PREP Studies included in the systematic review (N=91).**

| **First Author and publication year** | **Title of study** | **Program cycle** | **Study Design** | **Sample Size** | | **City, Country** |
| --- | --- | --- | --- | --- | --- | --- |
| Abrams-Downey et al. 2017 | “Risk Factors Associated With Sexually Transmitted Infections Among Pre-Exposure Prophylaxis Users In an Urban Multi-Clinic Healthcare System” [Open Forum Infect Dis](https://www.ncbi.nlm.nih.gov/pmc/articles/PMC5630945/). 2017 Fall; 4(Suppl 1): S668–S669. | 2013-2016 | Cohort study (open label) | 599 | | New York, USA |
| Ayedi et al. 2018 | "High incidence of STIs among MSM in PrEP without a significant increase." AIDS Research and Human Retroviruses 34 (Supplement 1): 259. | 2017-2018 | Cohort study | 100 | | Madrid, Spain |
| Bares et al. 2018 | "Acceptability and feasibility of a pharmacist-led pre-exposure prophylaxis program in the mid Western United States." Open Forum Infectious Diseases 5 (Supplement 1): S395. | 2016 - | Cohort study | 60 | | Omaha, Nebraska, USA |
| Bartovska et al. 2019 | "Pre-exposure prophylaxis, a new approach for HIV prevention: experience from the HIV Center of the Military University Hospital Prague." Epidemiologie, Mikrobiologie, Imunologie 69(1): 38-41. | 2017-2018 | Cohort study | 81 | | Prague, Czech Republic |
| Bavinton et al. 2019 | "Use of condoms for the prevention of sexually transmitted infections (STIS) among HIV preexposure prophylaxis (PREP) users." Sexually Transmitted Infections 95 (Supplement 1): A206. | 2016-2020 | Cohort study | 9733 | | Sydney, Australia |
| Berto et al. 2019 | "First results after 52 weeks of informal PrEP use in a cohort of MSM in Southern Spain." HIV Medicine 20 (Supplement 9): 241. | 2018 | Cohort study | 167 | | Seville, Spain |
| Beymer et al. 2018 | “Does HIV pre-exposure prophylaxis use lead to a higher incidence of sexually transmitted infections? A case-crossover study of men who have sex with men in Los Angeles, California.” Sexually Transmitted Infections vol. 94,6 (2018): 457-462. . | 2015-2016 | Routine Implementation | 275 | | Los Angeles, USA |
| Bhatia et al. 2018 | “Outcomes of Preexposure Prophylaxis Referrals From Public STI Clinics and Implications for the Preexposure Prophylaxis Continuum,” Sexually Transmitted Diseases Jan;45(1):50-55. | 2015-2016 | Routine Implementation | 137 | | Chicago, USA |
| Bristow et al. 2018 (Unpublished data) | Sexually transmitted infections and adherence to PrEP | 2018 | Cohort study (open label) | 394 | | California, USA |
| Carneiro et al. 2018 (Unpublished data) | Community Health Centre PrEP Program, New York State | 2018 | Routine implementation | 3820 | | New York, USA |
| Celum et al. 2020 | “Incentives conditioned on tenofovir levels to support PrEP adherence among young South African women: a randomized trial.” Journal of the International AIDS Society vol. 23,11 (2020): e25636. doi:10.1002/jia2.25636 | 2018 | Cohort study (open label) | 3000 | | Kenya, South Africa |
| Clement et al. 2020 | "Outcomes for PrEP users in the Southern U.S.: Persistence and STIs." AIDS Research and Human Retroviruses 34 (Supplement 1): 258. | 2013-2018 | Routine Implementation | 271 | | Durham, USA |
| Colby et al. 2018 | "$1 a day PrEP: Sustainable delivery of preexposure prophylaxis by the PrEP-30 program in Bangkok, Thailand." AIDS Research and Human Retroviruses 34 (Supplement 1): 270. | 2014-2018 | Routine Implementation | 1500 | | Thailand |
| Coleman et al. 2016 (Unpublished data) | Evaluation of patient engagement and health promotion in a community health PrEP Program | 2015-2016 | Routine Implementation | 193 | | USA |
| Connolly et al., 2020 | "Outcomes of a PrEP Demonstration Project with LGBTQ Youth in a Community-Based Clinic Setting with Integrated Gender-Affirming Care." Transgender Health 5(2): 75-79. | 2017-2018 | Demonstration project | 50 | | Detroit, Michigan, USA |
| Deborde et al. 2019 | "High prevalence of Mycoplasma genitalium infection and macrolide resistance in patients enrolled in HIV pre-exposure prophylaxis program." Medecine et Maladies Infectieuses 49(5): 347-349. | 2016-2017 | Cohort study | 88 | | France |
| Delany-Moretlwe et al. 2019 | "High curable STI prevalence and incidence among young African women initiating PrEP in HPTN 082." Sexually Transmitted Infections 95 (Supplement 1): A60-A61. | 2015-2017 | Cohort study | 412 | | Cape Town, Johannesburg in South Africa and Harare, Zimbabwe |
| Eakle et al. 2017 | “HIV pre-exposure prophylaxis and early antiretroviral treatment among female sex workers in South Africa: Results from a prospective observational demonstration project” PLOS Medicine 14(11): e1002444. | 2015-2017 | Cohort study (open label) | 219 | | South Africa |
| Eubanks et al. 2020 | "Reaching a Different Population of MSM in West Africa With the Integration of PrEP Into a Comprehensive Prevention Package (CohMSM-PrEP ANRS 12369-Expertise France)." Journal of Acquired Immune Deficiency Syndromes: JAIDS 85(3): 292-301. | 2016-2017 | Cohort Study | 524 | | Mali, Cote d'Ivoire, Burkina Faso, and Togo. |
| Feta et al. 2017 | “High Rate of Asymptomatic Bacterial Sexually Transmitted Infections (STIs) in Men who Have Sex with Men on Pre Exposure Prophylaxis (PrEP).” Open Forum Infectious Diseases vol. 4,Suppl 1 S669. | 2016 | Routine Implementation | 202 | | France |
| Freeborn et al. | “Sexual Risk Behavior in Men Who Have Sex with Men in an Era of Pre Exposure Prophylaxis (PrEP) for HIV Prevention” Dissertation research | 2014-2015 | Cohort study (open label) | 525 | | USA |
| Girometti et al. 2018 | "Evolution of a pre-exposure prophylaxis (PrEP) service in a community-located sexual health clinic: concise report of the PrEPxpress." Sexual Health 15(6): 598-600. | 2017-2018 | Routine Implementation | 1700 | | UK |
| Grant et al. 2014. | “Uptake of pre-exposure prophylaxis, sexual practices, and HIV incidence in men and transgender women who have sex with men: a cohort study”. The Lancet Infectious Diseases, 14 (9): P820-829. | 2011-2012 | Cohort study (open label) | 1225 | | USA |
| Green et al. 2018. | Prepped 4 PrEP! KP-led services in Vietnam. Paper presented at: The 2^nd^ Asia-Pacific Regional Consultation on PrEP Implementation; 2018 Jan 16; Bangkok, Thailand. Powerpoint presentation available from <https://www.prevention-trcarc.org/files/10.Kimberly%20Green-%20Prepped%204%20PrEP!.pdf> | 2018 | Routine Implementation | 636 | | Vietnam |
| Greenwald et al. 2019 | "Cohort profile: l'Actuel Pre-Exposure Prophylaxis (PrEP) Cohort study in Montreal, Canada." BMJ Open 9(6): e028768. | 2013-2018 | Cohort Study | 1551 | | Canada |
| Grinsztejn et al. 2018 | “Retention, engagement, and adherence to pre-exposure prophylaxis for men who have sex with men and transgender women in PrEP Brasil: 48 week results of a demonstration study” The Lancet HIV Mar; 5(3):e136-e145. | 2014-2016 | Demonstration project | 450 | | Brazil |
| Gulick et al. 2017 | “Phase 2 Study of the Safety and Tolerability of Maraviroc-Containing Regimens to Prevent HIV Infection in Men Who Have Sex With Men (HPTN 069/ACTG A5305).” The Journal of Infectious Diseases vol. 215,2: 238-246. | 2012-2014 | RCT (placebo-controlled) | 406 | | USA |
| Hamed et al. 2018 | "Incidence of sexually transmitted infections (STIS) in patients on preexposure prophylaxis (PREP)." Open Forum Infectious Diseases 5 (Supplement 1): S462. | 2016-2018 | cohort study | 74 | | New Jersey, USA |
| Hechter et al. 2018 | “Healthcare Utilization And STI Incidence In Young Men On Pre-Exposure Prophylaxis (PrEP) Compared To Young Men Who Are Not On PrEP: The PrEPARE Study” Presentation at the 2018 International AIDS Conference, Amsterdam, Netherlands | 2014-2016 | Routine Implementation | 304 | | USA |
| Golub et al. 2016 | “STI data from community-based prep implementation suggest changes to CDC guidelines” Presentation at the 2016 Conference on Retroviruses and Opportunistic Infections (CROI) | 2012-2016 | Cohort study (open label) | 280 | | USA |
| Hevey et al. 2018 | “PrEP Continuation, HIV and STI Testing Rates, and Delivery of Preventive Care in a Clinic-Based Cohort.” AIDS education and prevention : official publication of the International Society for AIDS Education vol. 30,5 (2018): 393-405. | 2010-2016 | Routine Implementation | 116 | | USA |
| Hightow-Weidman et al. 2019 | Hightow-Weidman, Lisa B et al. “Incidence and Correlates of Sexually Transmitted Infections Among Black Men Who Have Sex With Men Participating in the HIV Prevention Trials Network 073 Preexposure Prophylaxis Study.” Clinical Infectious Diseases: an official publication of the Infectious Diseases Society of America vol. 69,9: 1597-1604. | 2013-2014 | Cohort study (open label) | 226 | | USA |
| Hojilla et al. 2017 | Optimizing the Delivery of HIV Pre-Exposure Prophylaxis (PrEP): An Evaluation of Risk Compensation, Disengagement, and the PrEP Cascade. (dissertation research) | 2014-2015 | Routine Implementation | 344 | | USA |
| Hoornenborg et al. 2018 | “Men who have sex with men more often chose daily than event-driven use of pre-exposure prophylaxis: baseline analysis of a demonstration study in Amsterdam.” Journal of the International AIDS Society vol. 21,3: | 2015-2016 | Demonstration project | 376 | | Amsterdam |
| Hosek et al. 2017 | “Safety and Feasibility of Antiretroviral Preexposure Prophylaxis for Adolescent Men Who Have Sex With Men Aged 15 to 17 Years in the United States.” JAMA Pediatrics vol. 171,11: 1063-1071. | 2013-2014 | Demonstration project | 78 | | USA |
| Hoth et al. 2019 | Hoth, A. B., C. Shafer, D. B. Dillon, R. Mayer, G. Walton and M. E. Ohl (2019). "Iowa TelePrEP: A Public-Health-Partnered Telehealth Model for HIV Pre-Exposure Prophylaxis (PrEP) Delivery in a Rural State." Sexually Transmitted Diseases 30: 30. | 2017-2018 | Cohort study | 91 | | Iowa, USA |
| Iskandar 2018 | “Uptake of PrEP services among MSM in Malaysia: preliminary findings from myPrEP demonstration project” presentation at the Australian HIV & AIDS Conference, 26 September 2018. | 2018 | Demonstration project | 300 | | Malaysia |
| Kaewpoowat et al 2019 | "Prep acceptability, uptake, and adherence among young men who have sex with men and transgender women in PrEP demonstration project, Chiang Mai, Thailand." Open Forum Infectious Diseases 6 (Supplement 2): S462-S463. | 2015-2017 | Cohort study | 105 | | Chiangmai, Thailand |
| Karkashadze et al. 2019 | "No new HIV infections, but high incidence of syphilis among Pre-exposure Prophylaxis (PrEP) users in Georgia." HIV Medicine 20 (Supplement 9): 241. | 2017-2019 | Cohort study | 154 | | Georgia, USA |
| Khosropour et al. 2020. | “A Pharmacist-Led, Same-Day, HIV Pre-Exposure Prophylaxis Initiation Program to Increase PrEP Uptake and Decrease Time to PrEP Initiation.” AIDS patient care and STDs vol. 34,1 (2020): 1-6. | 2018-2019 | Cohort study | 69 | | Jackson, Mississippi, USA |
| Kinuthia et al. 2019 | “Pre-exposure prophylaxis uptake and early continuation among pregnant and post-partum women within maternal and child health clinics in Kenya: results from an implementation programme." The Lancet HIV 7(1): e38-e48. | 2017-2018 | Routine implementation | 2030 | | Kenya |
| Knapper at al.2018 | “Comorbidity, polypharmacy and renal impairment: The experience of managing a PrEP cohort in an integrated sexual health service setting” Presentation at the 4th Joint Conference of the British HIV Association (BHIVA) with the British Association for Sexual Health and HIV (BASHH), Edinburgh, UK, 17-20 April 2018, Edinburgh, UK. | 2017 | Routine Implementation | 74 | | UK |
| Kung et al. 2018 | "HIV pre-exposure prophylaxis (PrEP) implementation at Silom Community Clinic in Bangkok, Thailand, 2016-2018." Open Forum Infectious Diseases 5 (Supplement 1): S394-S395. | 2016-2018 | Cohort study | 192 | | Bangkok,  Thailand |
| Kwan et al. 2018 | Adherence of MSM participating in a partially self-financed pilot PrEP project and its association with behavioural risk profiles." Journal of the International AIDS Society 21 (Supplement 8): 29-30. | 2017-2018 | Cohort study | 71 | | Hongkong |
| Lal et al. 2017 | “Medication adherence, condom use and sexually transmitted infections in Australian preexposure prophylaxis users” AIDS. Jul 31;31(12):1709-1714. | 2014-2015 | Demonstration project | 114 | | Australian |
| Lalley-Chareczko et al 2018 | Delivery of TDF/FTC for Pre-exposure Prophylaxis to Prevent HIV-1 Acquisition in Young Adult Men Who Have Sex With Men and Transgender Women of Color Using a Urine Adherence Assay | 2015-2016 | Cohort study (open label) | 50 | | Philadelphia, Pennsylvania, USA |
| Leal Dos Santos et al. 2019 | "The Good and Bad of PrEP: A 14 month follow up on awareness, adherence, efficacy and sexually transmitted diseases at Hospital De Curry Cabral." HIV Medicine 20 (Supplement 9): 244. | 2018-2019 | Cohort study | 297 | | Portugal |
| Liu et al. 2015 | - “Adherence, sexual behaviour and HIV/STI incidence among men who have sex with men and transgender women in the US PrEP demonstration (Demo) project”. June 2015. Journal of the International AIDS Society Conference proceedings. | 2012-2015 | Routine Implementation | 557 | | San Francisco, Washington DC and Miami, USA |
| Liu et al. 2016 | “Prep initiation and early adherence among young MSM and transgender women in Chicago in the enhancing PrEP in community (EPIC) study”. AIDS Research and Human Retroviruses, 2016, 32, 81. Conference presentation | 2015-2016 | Open-label RCT | 121 | | USA |
| Lopez et al. 2019 | "Implementation of pre-exposure prophylaxis at a community pharmacy through a collaborative practice agreement with San Francisco Department of Public Health." Journal of the American Pharmacists Association: JAPhA 60(1): | 2016-2019 | Demonstration project | 51 | | San Francisco, USA |
| Marcus et al. 2016 | “Preexposure Prophylaxis for HIV Prevention in a Large Integrated Health Care System: Adherence, Renal Safety, and Discontinuation.” Journal of Acquired Immune Deficiency Syndromes vol. 73,5 (2016): 540-546. | 2012-2014 | Cohort study (open label) | 972 | | USA |
| Marcus et al. 2013 | “No evidence of sexual risk compensation in the iPrEx trial of daily oral HIV preexposure prophylaxis.” PloS One vol. 8,12 e81997. 18 Dec. 2013, doi:10.1371/journal.pone.0081997 | 2007-2009 | RCT (placebo-controlled) | 2499 | | 11 international sites (Peru, Ecuador, South Africa, Brazil, Thailand & USA) |
| Mboup et al. 2018 | "Early antiretroviral therapy and daily pre-exposure prophylaxis for HIV prevention among female sex workers in Cotonou, Benin: a prospective observational demonstration study." Journal of the International AIDS Society 21(11). | 2014-2016 | Cohort study | 422 | | Benin |
| McCormack et al. 2016 | “Pre-exposure prophylaxis to prevent the acquisition of HIV-1 infection (PROUD): effectiveness results from the pilot phase of a pragmatic open-label randomised trial” The [Lancet](https://www.ncbi.nlm.nih.gov/pmc/articles/PMC4700047/). Jan 2; 387(10013): 53–60 | 2012-2014 | Open-label RCT | 544 | | England |
| Migueres et al. 2020 | "No evidence of sexual transmission of HEV among individuals using HIV pre-exposure prophylaxis." Journal of Viral Hepatitis 27(12): 1495-1501. | 2016-2019 | Cohort study | 135 | | France |
| Milam et al. 2019 | "Sexual risk compensation in a pre-exposure prophylaxis demonstration study among individuals at risk of HIV." JAIDS, Journal of Acquired Immune Deficiency Syndromes 80(1): e9-e13. | 2013 | RCT | 398 | | USA |
| Mizushima et al.  (Unpublished data) | “A clinical study to determine whether HIV infection rates can be reduced if high-risk individuals take anti-viral medicine on a daily basis.” | 2018 | Demonstration project | 591 | | Japan |
| Molina et al. 2015 | “On-Demand Preexposure Prophylaxis in Men at High Risk for HIV-1 Infection” New England Journal of Medicine; 373:2237-2246 | 2012-2014 | RCT (placebo-controlled) | 400 | | France and Canada |
| Montano et al. 2019 | “Changes in Sexual Behavior and STI Diagnoses Among MSM Initiating PrEP in a Clinic Setting.” AIDS & Behavior vol. 23,2: 548-555. | 2014-2017 | Routine implementation | 376 | | USA |
| Morgan et al. 2019 | PrEP Use and Sexually Transmitted Infections Are Not Associated Longitudinally in a Cohort Study of Young Men Who Have Sex with Men and Transgender Women in Chicago." AIDS & Behavior 24(5): 1334-1341. | 2007-2018 | Cohort study | 744 | | USA |
| Morgat et al. 2020 | Number, type and cost of microbiological tests during HIV Pre-Exposure Prophylaxis: The experience of a French hospital." Medecine et Maladies Infectieuses 20: 20. | 2016-2019 | Routine implementation | 135 | | France |
| Morris-Haris et al 2019. | "Lower incidence rates of Neisseria gonorrhoeae and Chlamydia trachomatis in pre-exposure prophylaxis patients over fifty years old than in younger quartiles." Open Forum Infectious Diseases 6 (Supplement 2): S214. | 2016-2019 | Routine implementation | 201 | | USA |
| Moussa 2018 (Unpublished data) | Assessing the feasibility of PrEP and the acceptability of key populations | 2018 | Demonstration project | 400 | | Morocco |
| Mugwanya et al. 2013 | “Sexual behaviour of heterosexual men and women receiving antiretroviral pre-exposure prophylaxis for HIV prevention: a longitudinal analysis” The Lancet Infectious Diseases Dec; 13(12): 1021-8. | 2011-2012 | Cohort study (open label) | 3024 | | Kenya and Uganda |
| Mullick. 2018. (Unpublished data) | Project PrEP | 2018 | Demonstration project | -- | | South Africa |
| Myers et al. 2020 | "Adherence to PrEP Among Young Men Who Have Sex With Men Participating in a Sexual Health Services Demonstration Project in Alameda County, California." Journal of Acquired Immune Deficiency Syndromes: JAIDS 81(4): 406-413. | 2014-2015 | Demonstration project | 257 | | USA |
| Nguyen et al. 2018 | “Incidence of sexually transmitted infections before and after preexposure prophylaxis for HIV” AIDS. 2018 Feb 20;32(4):523-530 | 2010-2015 | Cohort study (open label) | 109 | | Canada |
| Noret et al. 2018 | “Daily or on-demand oral tenofovir disoproxil fumarate/emtricitabine for HIV pre-exposure prophylaxis: experience from a hospital-based clinic in France” AIDS, Sep 24; 32(15): 2161-2169. | 2015-2017 | Cohort study (open label) | 1049 | | France |
| O'Byrne et al. 2020 | Immediate PrEP after PEP: Results from an Observational Nurse-Led PEP2PrEP Study." Journal of the International Association of Providers of AIDS Care 19: 2325958220939763. | 2018 | Routine implementation | 30 | | Canada |
| Phanuphak et al. 2018. | “Princess PrEP program: The first key population-led model to deliver pre-exposure prophylaxis to key populations by key populations in Thailand” Sexual Health. Nov;15(6):542-555. | 2016-2017 | Demonstration project | 1697 | | Thailand |
| Refugio et al. 2019 \ | "PrEPTECH: a telehealth-based initiation program for HIV pre-exposure prophylaxis in young men of color who have sex with men. A pilot study of feasibility." JAIDS, Journal of Acquired Immune Deficiency Syndromes 80(1): 40-45.2017 | 2016-2017 | Cohort | 25 | | USA |
| Roth et al. 2020 | "Integrating HIV pre-exposure prophylaxis with community-based syringe services for women who inject drugs: Results from the Project SHE demonstration study." Journal of Acquired Immune Deficiency Syndromes: JAIDS 28: 28. | 2018-2019 | Cohort | 96 | | USA |
| Ryan et al. 2020 | Trends in HIV and STI testing among gay bisexual and other men who have sex with men following rapid scale-up of PrEP in Victoria, Australia. Sexually Transmitted Diseases, 47 (8): 516-524. | 2015-2016 | Routine implementation | 4265 | Australia | |
| Sarr et al. 2020 | "Uptake, retention, and outcomes in a demonstration project of pre-exposure prophylaxis among female sex workers in public health centers in Senegal." International Journal of STD and AIDS 31(11): 1063-1072.2016 | 2015-2016 | Demonstration project | 267 | | Senegal |
| Schumacher et al. 2019 | STI Screening among Gay, Bisexual and Other Men who Have Sex with Men Prescribed PrEP in Baltimore City, Maryland." Clinical Infectious Diseases 25: 25. | 2015-2018 | Routine implementation | 290 | | USA |
| Songtaweesin et al. 2020 | "Youth-friendly services and a mobile phone application to promote adherence to pre-exposure prophylaxis among adolescent men who have sex with men and transgender women at-risk for HIV in Thailand: a randomized control trial." Journal of the International AIDS Society 23 Suppl 5: e25564. | 2018-2019 | RCT | 200 | | Thailand |
| St Clair et al. 2019 | "Adherence and sexually transmitted infections among MSM receiving care in a community based HIV prep clinic in the deep south." Sexually Transmitted Infections 95 (Supplement 1): A250. | 2016-2018 | Routine implementation | 119 | | USA |
| Streeck et al. 2019 | "Prospective, multicenter study to assess point prevalence, incidence and recurrence of sexually transmitted infections in men who have sex with men in Germany: BRAHMS study." Journal of the International AIDS Society. Conference: 10th IAS Conference on HIV Science. Mexico. 22(Supplement 5). | 2018-2019 | Cohort study | 1000 | | Germany |
|  |  |  |  |  | |  |
| Subedar 2018 (Unpublished data) | National Department of Health PrEP Program | 2018 | Cohort study (open label) | 200 | | South Africa |
| Tabatabavakili et al. 2019 | "Incidence of hepatitis C virus infections among users of HIV pre-exposure prophylaxis in a large academic centre in Toronto, Canada." Hepatology v70 suppl.1 2019 70 (Supplement 1): 182A-183A. | 2012-2019 | Routine implementation | 344 | | Canada |
| Tiberio et al. 2015 | “Prepared: Implementation of a pre-exposure prophylaxis (PrEP) program in a hospital-based HIV clinic” Proceedings of the 8th Annual Conference on the Science of Dissemination and Implementation: Washington, DC, 14-15 December 2015 | 2014 | Demonstration project | 33 | | USA |
| Torres et al. 2018 (Unpublished data) | Medical Centre PrEP Program, New York | 2018 | Routine implementation | -- | | New York, USA |
| Van Praet et al. 2019. | "Mycoplasma genitalium acquisition and macrolide resistance after initiation of HIV pre-exposure prophylaxis in men who have sex with men." Sexually Transmitted Infections 96(6): 396-398. | 2017-2019 | Routine implementation | 131 | | Belgium |
| Veloso et al. 2019 | "Safety, early continuation and adherence of same day PrEP initiation among MSM and TGW in Brazil, Mexico and Peru: The ImPrEP Study." Journal of the International AIDS Society. Conference: 10th IAS Conference on HIV Science. Mexico. 22(Supplement 5). | 2018-2019 | Demonstration project | 3257 | | Brazil, Mexico, Peru |
| Volk et al. 2015 | “No New HIV Infections With Increasing Use of HIV Preexposure Prophylaxis in a Clinical Practice Setting.” Clinical infectious diseases: an official publication of the Infectious Diseases Society of America vol. 61,10 (2015): 1601-3. | 2012-2015 | Routine Implementation | 657 | | USA |
| Vuylsteke 2018. (Unpublished data) | Reaching a Different Population of MSM in West Africa With the Integration of PrEP Into a Comprehensive Prevention Package (CohMSM-PrEP ANRS 12369—Expertise France) | 2018 | Demonstration project | 500 | | West Africa |
| Wang et al. 2018 | InterPrEP: internet-based pre-exposure prophylaxis with generic tenofovir disoproxil fumarate/emtrictabine in London - analysis of pharmacokinetics, safety and outcomes, HIV Med.  Jan;19(1):1-6. | 2016 | Demonstration project | 293 | | England |
| Wahome et al. 2019 | Assessment of PrEP eligibility and uptake among at-risk MSM participating in a HIV-1 vaccine feasibility cohort in coastal Kenya. Wellcome Open Research. 4:138. | 2005-2011 | Cohort study (open label) | 449 | | Kenya |
| Wu et al. 2020 | Syphilis Acquisition And Dosing Schedule For Pre-Exposure Prophylaxis (PrEP) Users In Taiwan PrEP Demonstration Project, Poster presentation at 22nd International AIDS Conference (AIDS 2018), Amsterdam, Netherlands, 23-27 July 2018 | 2016-2017 | Demonstration project | 302 | | Taiwan, China |
| Zablotska et al. 2018 | “Expanded HIV pre-exposure prophylaxis (PrEP) implementation in communities in New South Wales, Australia (EPIC-NSW): design of an open label, single arm implementation trial.” BMC public health vol. 18,1 210. 2 | 2016-2020 | Cohort study (open label) | 3700 | | Australia |
| Zablotska et al. 2020 | "High Adherence to HIV Pre-exposure Prophylaxis and No HIV Seroconversions Despite High Levels of Risk Behaviour and STIs: The Australian Pr Demonstration Study PrELUDE." AIDS & Behavior 23(7): 1780-1789. | 2014-2016 | Cohort study | 323 | | Australia |

**Supplemental File 1**

PRISMA Checklist

| **Section/topic** | **#** | **Checklist item** | **Reported on page #** |
| --- | --- | --- | --- |
| **TITLE** | | |  |
| Title | 1 | Identify the report as a systematic review, meta-analysis, or both. | 1 |
| **ABSTRACT** | | |  |
| Structured summary | 2 | Provide a structured summary including, as applicable: background; objectives; data sources; study eligibility criteria, participants, and interventions; study appraisal and synthesis methods; results; limitations; conclusions and implications of key findings; systematic review registration number. | 2 |
| **INTRODUCTION** | | |  |
| Rationale | 3 | Describe the rationale for the review in the context of what is already known. | 3 |
| Objectives | 4 | Provide an explicit statement of questions being addressed with reference to participants, interventions, comparisons, outcomes, and study design (PICOS). | 3 |
| **METHODS** | | |  |
| Protocol and registration | 5 | Indicate if a review protocol exists, if and where it can be accessed (e.g., Web address), and, if available, provide registration information including registration number. | 4 |
| Eligibility criteria | 6 | Specify study characteristics (e.g., PICOS, length of follow-up) and report characteristics (e.g., years considered, language, publication status) used as criteria for eligibility, giving rationale. | 4 |
| Information sources | 7 | Describe all information sources (e.g., databases with dates of coverage, contact with study authors to identify additional studies) in the search and date last searched. | 4 |
| Search | 8 | Present full electronic search strategy for at least one database, including any limits used, such that it could be repeated. | 4 (referenced published paper) and supplemental file 3 |
| Study selection | 9 | State the process for selecting studies (i.e., screening, eligibility, included in systematic review, and, if applicable, included in the meta-analysis). | 4 |
| Data collection process | 10 | Describe method of data extraction from reports (e.g., piloted forms, independently, in duplicate) and any processes for obtaining and confirming data from investigators. | 4 |
| Data items | 11 | List and define all variables for which data were sought (e.g., PICOS, funding sources) and any assumptions and simplifications made. | 4 |
| Risk of bias in individual studies | 12 | Describe methods used for assessing risk of bias of individual studies (including specification of whether this was done at the study or outcome level), and how this information is to be used in any data synthesis. | 4 |
| Summary measures | 13 | State the principal summary measures (e.g., risk ratio, difference in means). | 5 |
| Synthesis of results | 14 | Describe the methods of handling data and combining results of studies, if done, including measures of consistency (e.g., I^2^) for each meta-analysis. | 5 |

**Supplemental File 2**

Opportunities and costs for controlling sexually transmitted infections among populations using pre-exposure prophylaxis for HIV: Search methodology and search results

2020 Update

# Search methodology

The search strategies described at <https://doi.org/10.17037/DATA.00001778> were updated on 08 December 2020. No changes were made to the search strategies.

The databases searched were:

1. OvidSP Medline ALL, 1946 to December 04, 2020
2. OvidSP Embase, 1974 to 2020 December 07
3. OvidSP Global Health, 1910 to 2020 week 48
4. OvidSP EconLit, 1886 to November 26, 2020
5. EBSCO CINAHL Plus, complete database
6. EBSCO Africa-Wide Information, complete database
7. Web of Science Core Collection, which included:
   1. Science Citation Index Expanded, 1970 – 07 December 2020
   2. Social Sciences Citation Index, 1970– 07 December 2020
   3. Arts & Humanities Citation Index, 1975– 07 December 2020
   4. Conference Proceedings Citation Index- Science, 1990– 07 December 2020
   5. Conference Proceedings Citation Index- Social Science & Humanities, 1990– 07 December 2020
   6. Emerging Sources Citation Index, 2015– 07 December 2020
8. VHL LILACS, complete database

The OvidSP Northern Light Life Sciences Conference Abstracts database was not searched as this was no longer available.

# Search results

Search results were uploaded to EndNote X9 and deduplicated. using a technique developed at the University of Leeds.^[[1]](#footnote-1)^ The results were deduplicated against the results retrieved in 2018, so only results uploaded to the databases since the first search were retained for screening. Number of results pre- and post- deduplication are listed in the table below.

| Database name | EndNote import order | Number of references before deduplication | Number of references after deduplication |
| --- | --- | --- | --- |
| Medline | 1 | 957 | 431 |
| Embase | 2 | 2168 | 531 |
| Global Health | 3 | 990 | 190 |
| EconLit | N/A | 0 | 0 |
| CINAHL Plus | 4 | 433 | 40 |
| Africa-Wide Information | 5 | 95 | 5 |
| Web of Science | 6 | 1188 | 117 |
| LILACS | 7 | 5 | 4 |
| **Total** |  | 5836 | 1318 |

# Search strategies

Updated search strategies are available on the London School of Hygiene & Tropical Medicine data repository at <https://doi.org/10.17037/DATA.00001989>.

**Supplemental File 3: Survey for PrEP implementers**

**COUNTRY/HEALTH JURISDICTION:**

**Completed by:**

**Name:**

**Affiliation:**

**Contact information (email):**

| **Overall goal:**  To evaluate PrEP implementation and its impact on STI services and prevention.  **Specific review objective:**  To collect baseline data on incidence and prevalence of STIs and provision of STI services |
| --- |

1. **PrEP services**
2. What PrEP guidelines does the country use? *(please share a .pdf, preferably in English)*
3. When were these PrEP guidelines most recently issued?
4. What are the populations provided with PrEP?
5. How frequently are PrEP users seen in the clinic for HIV testing?
6. In addition to HIV testing, is STI screening being done?
7. Is STI screening integrated into the same service as for PrEP? Or do clients need to attend a different service provider for STI screening?
8. Does your PrEP services provide vaccination (e.g. for Hepatitis A/B, HPV)?
9. **STI screening and treatment guidelines**
10. Do you have an existing STI screening and treatment policy for your country? Please provide a copy.
11. Do you have an STI screening and treatment policy for PrEP users and/or key populations? Please provide a copy.
12. For your PrEP program, could you estimate the following STI cascade:

**PrEP Users**

Year of data:__________

Total number of PrEP users: ____________

|  | Eligible for screening | Offered screening | Received test | Tested positive | Successfully treated |
| --- | --- | --- | --- | --- | --- |
| Chlamydia |  |  |  |  |  |
| LGV |  |  |  |  |  |
| Gonorrhoea |  |  |  |  |  |
| Syphilis |  |  |  |  |  |
| M. genitalium |  |  |  |  |  |
| Hepatitis A |  |  |  |  |  |
| Hepatitis B |  |  |  |  |  |
| Hepatitis C |  |  |  |  |  |
| Herpes |  |  |  |  |  |

1. **INCIDENCE data (case reporting of STIs) by population – e.g. MSM, SW, young women**
2. Please provide any STI incidence data for PrEP users. Alternatively, fill out the table below.

**PrEP users – new STI cases in a year.**

If different time period (e.g. 6 months): please specify _______________

Year of incidence data: ____________

Total number of total PrEP users: ____________

Total number of MSM PrEP users: ____________

Total number of Sex worker PrEP users: ____________

Total number of Transgender PrEP users: ____________

Total number of HIV serodiscordant couples PrEP users: ____________

Total number of Adolescent PrEP users: ____________

Total number of Other PrEP users: ____________

|  | Total  n ^#^ | MSM  n ^#^ | Sex workers  n ^#^ | Transgender  n ^#^ | HIV serodiscordant couples  n ^#^ | Adolescents  n ^#^ | Other  Specify:  n ^#^ |
| --- | --- | --- | --- | --- | --- | --- | --- |
| Chlamydia |  |  |  |  |  |  |  |
| LGV |  |  |  |  |  |  |  |
| Gonorrhoea |  |  |  |  |  |  |  |
| Syphilis |  |  |  |  |  |  |  |
| M. genitalium |  |  |  |  |  |  |  |
| Hepatitis A |  |  |  |  |  |  |  |
| Hepatitis B |  |  |  |  |  |  |  |
| Hepatitis C |  |  |  |  |  |  |  |
| Herpes |  |  |  |  |  |  |  |

^#^ n = number of new cases

**Non-PrEP users* – new STI cases in a year.**

* This may be a comparable population attending the STI services or a comparable community population

If different time period (e.g. 6 months): please specify _______________

Year of incidence data: ____________

Total number of total study population: ____________

Total number of MSM: ____________

Total number of Sex workers: ____________

Total number of Transgender: ____________

Total number of HIV serodiscordant couples: ____________

Total number of Adolescent: ____________

Total number of Other: ____________

|  | Total  n^#^ | MSM  n^#^ | Sex workers  n^#^ | Transgender  n^#^ | HIV serodiscordant couples  n^#^ | Adolescents  n^#^ | Other  Specify:  n^#^ |
| --- | --- | --- | --- | --- | --- | --- | --- |
| Chlamydia |  |  |  |  |  |  |  |
| LGV |  |  |  |  |  |  |  |
| Gonorrhoea |  |  |  |  |  |  |  |
| Syphilis |  |  |  |  |  |  |  |
| M. genitalium |  |  |  |  |  |  |  |
| Hepatitis A |  |  |  |  |  |  |  |
| Hepatitis B |  |  |  |  |  |  |  |
| Hepatitis C |  |  |  |  |  |  |  |
| Herpes |  |  |  |  |  |  |  |

^#^ n = number of STI cases, N = total number tested

1. **PREVALENCE data from surveys for specific populations**
2. Please provide any STI prevalence data for PrEP users. Alternatively, fill out the table below.

**PrEP users – STI cases detected (e.g. at baseline)**

Year of prevalence data: ____________

|  | Total  n/N^#^ | MSM  n/N^#^ | Sex workers  n/N^#^ | Transgender  n/N^#^ | HIV serodiscordant couples  n/N^#^ | Adolescents  n/N^#^ | Other  Specify:  n/N^#^ |
| --- | --- | --- | --- | --- | --- | --- | --- |
| Chlamydia |  |  |  |  |  |  |  |
| LGV |  |  |  |  |  |  |  |
| Gonorrhoea |  |  |  |  |  |  |  |
| Syphilis |  |  |  |  |  |  |  |
| M. genitalium |  |  |  |  |  |  |  |
| Hepatitis A |  |  |  |  |  |  |  |
| Hepatitis B |  |  |  |  |  |  |  |
| Hepatitis C |  |  |  |  |  |  |  |
| Herpes |  |  |  |  |  |  |  |

^#^ n = number of cases, N = total number tested

**Non-PrEP users* – STI cases detected (e.g. from baseline)**

* This may be a comparable population attending the STI services or a comparable community population

Year of prevalence data: ____________

|  | Total  n/N^#^ | MSM  n/N^#^ | Sex workers  n/N^#^ | Transgender  n/N^#^ | HIV serodiscordant couples  n/N^#^ | Adolescents  n/N^#^ | Other  Specify:  n/N^#^ |
| --- | --- | --- | --- | --- | --- | --- | --- |
| Chlamydia |  |  |  |  |  |  |  |
| LGV |  |  |  |  |  |  |  |
| Gonorrhoea |  |  |  |  |  |  |  |
| Syphilis |  |  |  |  |  |  |  |
| M. genitalium |  |  |  |  |  |  |  |
| Hepatitis A |  |  |  |  |  |  |  |
| Hepatitis B |  |  |  |  |  |  |  |
| Hepatitis C |  |  |  |  |  |  |  |
| Herpes |  |  |  |  |  |  |  |

^#^ n = number of STI cases, N = total number tested

1. **Risk behaviors of PrEP and non-PrEP users**

**PrEP Users**

Year of data: ________

|  | Total | MSM | Sex workers | Transgender | HIV serodiscordant couples | Adolescents | Other  Specify: |
| --- | --- | --- | --- | --- | --- | --- | --- |
| Number of sexual partners in last ____ months |  |  |  |  |  |  |  |
| Proportion always using condoms in last ____ months |  |  |  |  |  |  |  |

**Non-PrEP Users** (this may be from a comparable population attending the STI services or a comparable community population)

Year of data:____________

|  | Total | MSM | Sex workers | Transgender | HIV serodiscordant couples | Adolescents | Other  Specify: |
| --- | --- | --- | --- | --- | --- | --- | --- |
| Number of sexual partners in last ____ months |  |  |  |  |  |  |  |
| Proportion always using condoms in last ____ months |  |  |  |  |  |  |  |

1. **What are you screening for and how often?**

In PrEP services (whether from demonstration projects, or actual national programmes), do you screen for STIs?

| **Pathogen** | **Baseline assessment (Yes or No)** | **Frequency of STI testing (e.g. month 3, month 6)** | **Type of assay (e.g. nucleic acid testing, culture)** | **Type of specimen (anorectal, vaginal, urethral, urine, pharyngeal, serum)** | **Rapid test (Yes or No)** | **Test for antimicrobial resistance? (Yes or No)** |
| --- | --- | --- | --- | --- | --- | --- |
| Chlamydia |  |  |  |  |  |  |
| LGV |  |  |  |  |  |  |
| Gonorrhoea |  |  |  |  |  |  |
| Syphilis |  |  |  |  |  |  |
| M. genitalium |  |  |  |  |  |  |
| Hepatitis A |  |  |  |  |  |  |
| Hepatitis B |  |  |  |  |  |  |
| Hepatitis C |  |  |  |  |  |  |
| Herpes |  |  |  |  |  |  |

1. **COSTING DATA**
2. Are STI services free of charge for all PrEP users? (Yes/No)
3. Are STI drugs provided for free for PrEP users? (Yes/No)
4. Are lab test provided for free for PrEP users? (Yes/No)
5. If STI services are not free, what are the out of the pocket expenses by patients?
   1. Consultation fee
   2. STI drugs
   3. STI laboratory services
6. Are STI laboratory services available onsite (same building) as the PrEP clinic(s)?
7. What STI samples need to be referred to an offsite laboratory?
8. When referred, how long to receive the laboratory results?
9. What do you see as the major obstacles for provision of STI screening and treatment in the settings providing PrEP?
10. What do you see as the advantages of provision of STI services with PrEP services?
11. What could be done to help with provision of STI services with PrEP for comprehensive prevention services?

1. Deduplication method is published at <http://medhealth.leeds.ac.uk/download/2518/auhe_duplicate_checking_guide>. [↑](#footnote-ref-1)
